# Tunable Lattice Coupling of Multipole Plasmon Modes and Near-Field Enhancement in Closely Spaced Gold Nanorod Arrays

**DOI:** 10.1038/srep23159

**Published:** 2016-03-17

**Authors:** Yu Huang, Xian Zhang, Emilie Ringe, Mengjing Hou, Lingwei Ma, Zhengjun Zhang

**Affiliations:** 1 State Key Laboratory of New Ceramics and Fine Processing, School of Materials Science and Engineering, Tsinghua University, Beijing 100084, P. R. China; 2 Deparment of Materials Science and Nanoengineering & Laboratory for Nanophotonics, Rice University, 6100 Main Street, Houston, TX 77005, USA; 3 Key Laboratory of Advanced Materials (MOE), School of Materials Science and Engineering, Tsinghua University, Beijing 100084, P. R. China

## Abstract

Considering the nanogap and lattice effects, there is an attractive structure in plasmonics: closely spaced metallic nanoarrays. In this work, we demonstrate experimentally and theoretically the lattice coupling of multipole plasmon modes for closely spaced gold nanorod arrays, offering a new insight into the higher order cavity modes coupled with each other in the lattice. The resonances can be greatly tuned by changes in inter-rod gaps and nanorod heights while the influence of the nanorod diameter is relatively insignificant. Experimentally, pronounced suppressions of the reflectance are observed. Meanwhile, the near-field enhancement can be further enhanced, as demonstrated through surface enhanced Raman scattering (SERS). We then confirm the correlation between the near-field and far-field plasmonic responses, which is significantly important for maximizing the near-field enhancement at a specific excitation wavelength. This lattice coupling of multipole plasmon modes is of broad interest not only for SERS but also for other plasmonic applications, such as subwavelength imaging or metamaterials.

Metallic nanoparticles can undergo light-driven collective oscillations of the conduction electrons and support localized surface plasmon resonances (LSPRs)^1^. By virtue of being small, such particles are able to concentrate and guide light at the sub-wavelength scale^2^ and provide extremely large, localized near-field enhancement^3^. These unique properties benefit applications in a wide variety of fields such as plasmonic waveguiding^4^, chemical and biological sensing^5^,^6^, surface-enhanced spectroscopies^7^,^8^,^9^,^10^,^11^, to name a few.

Recently, periodic metallic nanostructures, i.e. plasmonic crystals, are of particular interest for these applications, as they can drastically improve the quality factor of LSPRs^12^ and provide further optimization of the optical response^13^,^14^. It is revealed that when the array period is commensurate with the excitation wavelength, the collective resonances can be generated by the diffractive coupling of individual LSPRs in the lattice^15^,^16^,^17^,^18^, of which the physical origin is attributed to the Fano interference^19^,^20^. On the other hand, subwavelength metallic arrays, also known as nanoplasmonic metamaterials, can support a guided mode below the diffraction limit of light, enabling subwavelength imaging, nanolasing and enhanced nonlinear effects^21^,^22^,^23^,^24^. Besides, strong near-field enhancement like electromagnetic hot spots is commonly obtained by narrowing the gaps between metallic nanostructures^25^,^26^. Considering these gap and lattice effects, there is an attractive structure: closely spaced metallic nanoarrays, which serves as an active plasmonic platform^27^,^28^,^29^,^30^.

Here we present a comprehensive study of both the near-field and far-field plasmonic properties for closely spaced gold nanorod arrays, demonstrating the lattice coupling of multipole plasmon modes both experimentally and theoretically. In particular, we observe a set of pronounced dips in the reflectance spectra with good tunability. When the array is excited at the resonance wavelength, the near-field response in terms of surface enhanced Raman scattering (SERS)^28^,^31^,^32^,^33^ can be further enhanced by nearly an order of magnitude. It is further revealed that the near-field and far-field responses correlate well with each other, which is of significant importance for maximizing the near-field enhancement at a specific excitation wavelength.

## Results

### Lattice coupling of multipole plasmon modes

**Figure 1:**
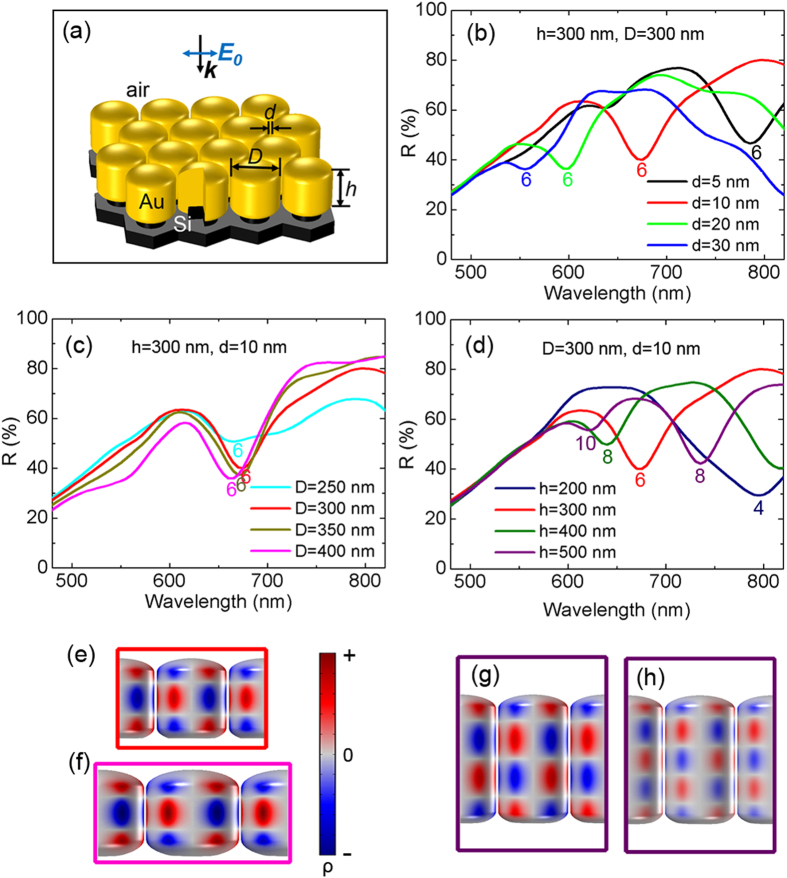
Lattice coupling of multipole plasmon modes. (**a**) Lattice structure of gold nanorod arrays in the simulation. The incident light is linearly polarized along the inter-rod axis. For simplicity, |***E***_***0***_*|* = 1 V/m. (**b**–**d**) FEM simulated reflectance spectra using the control variate method: (**b**) Varying *d* while keeping *h* = 300 nm and *D* = 300 nm; (**c**) Varying *D* while keeping *h* = 300 nm and *d* = 10 nm; (**d**) Varying *h* while keeping *D* = 300 nm and *d* = 10 nm. (**e–h**) Typical 3D surface charge distributions at the resonance wavelengths *λ* indicated by the reflection dips, demonstrating the lattice coupling of multipole plasmon modes: (**e**) *λ* = 675 nm, six-pole for array *h* = 300 nm, *D* = 300 nm, *d* = 10 nm; (**f**) *λ* = 665 nm, six-pole for array *h* = 300 nm, *D* = 400 nm, *d* = 10 nm; (**g**) *λ* = 735 nm, eight-pole for array *h* = 500 nm, *D* = 300 nm, *d* = 10 nm; (**h**) *λ* = 620 nm, ten-pole for *h* = 500 nm, *D* = 300 nm, *d* = 10 nm. Red and blue correspond to positive and negative charges, respectively.

The gold lattice structure on the silicon template in this study is schematically depicted in Fig. 1a, which is defined by the inter-rod gap *d*, the gold nanorod diameter *D* and its height *h*. To give a general idea of these geometry parameters’ influence on the optical response of the lattice, the reflectance *R* is calculated with 5 nm wavelength spacing by 3D finite element method (FEM) simulations using the control variate method (See Methods). As is shown in Fig. 1b–d, there are a series of pronounced reflection dips, indicating an efficient coupling of the arrays to the incident light and also different resonant plasmonic modes in the lattice. For these subwavelength periodic gold nanorod arrays, the reflection dips cannot be interpreted by Rayleigh anomalies in plasmonic Fano resonances^19^ or the fundamental dipole resonance of individual nanorods^15^,^29^.

To confirm the plasmon modes, 3D surface charge distributions are calculated by applying Gauss’ law during the simulation^25^,^26^. Considering the skin effect at visible frequency, the charge density *ρ* at the metal surface is nearly proportional to (*n*_*x*_*∙E*_*x*_ + *n*_*y*_*∙E*_*y*_ + *n*_*z*_*∙E*_*z*_), where ***n*** = (*n*_*x*_*, n*_*y*_*, n*_*z*_) is the outward normal vector of the metal surface and ***E*** = (*E*_*x*_, *E*_*y*_, *E*_*z*_) is the local electric field (See Methods). As a result, (*n*_*x*_*∙E*_*x*_ + *n*_*y*_*∙E*_*y*_ + *n*_*z*_*∙E*_*z*_) is used to indicate the surface charge density *ρ* in the process of 3D plasmon mapping. Typical surface charge distributions are plotted in Fig. 1e–h, demonstrating clearly the lattice coupling of multipole plasmon modes.

Seen from Supplementary Movie S1, the charge poles on gold nanorod surface alternate between negative and positive as the conduction electrons are driven by the oscillating electric field of incident light. The plotted transient surface charge is of the maximum polarization within one oscillation. Viewed from single nanorod, the mapping reveals the transverse multipole plasmon mode. Considering the inter-rod gap, these modes can also be assigned to the cavity modes^27^,^28^,^29^,^34^. For example, the six-pole plasmon mode (Fig. 1e,f) can be understood as the second-order cavity mode. Usually, cavity modes are found in metal-insulator-metal waveguide structures due to Fabry-Perot resonances in the longitudinal direction. The fundamental cavity mode corresponds to a dipole surface charge distributed antisymmetrically at each metal sidewall^35^,^36^,^37^. Our 3D plasmon mapping here offers a new insight into the higher order cavity modes coupled with each other in closely spaced plasmonic nanoarrays.

### Tunability of lattice coupling

As is shown in Fig. 1b–d, all the reflections dips are marked by the surface charge pole number of the corresponding single nanorod. Seen from Fig. 1a, the six-pole lattice coupling shifts to longer wavelength rapidly as *d* decreases from 30 nm to 5 nm, while the influence of *D* on the resonance position is relatively insignificant (Fig. 1c). By increasing *h*, higher-order multipole can be generated in the detecting wavelength range, accompanied by a pronounced redshift of the corresponding lower-order multipole modes (Fig. 1d). The great tunability of the lattice coupling of multipole palsmon modes offers a promising candidate for plasmonic applications.

### Gold nanoarray fabrication

**Figure 2:**
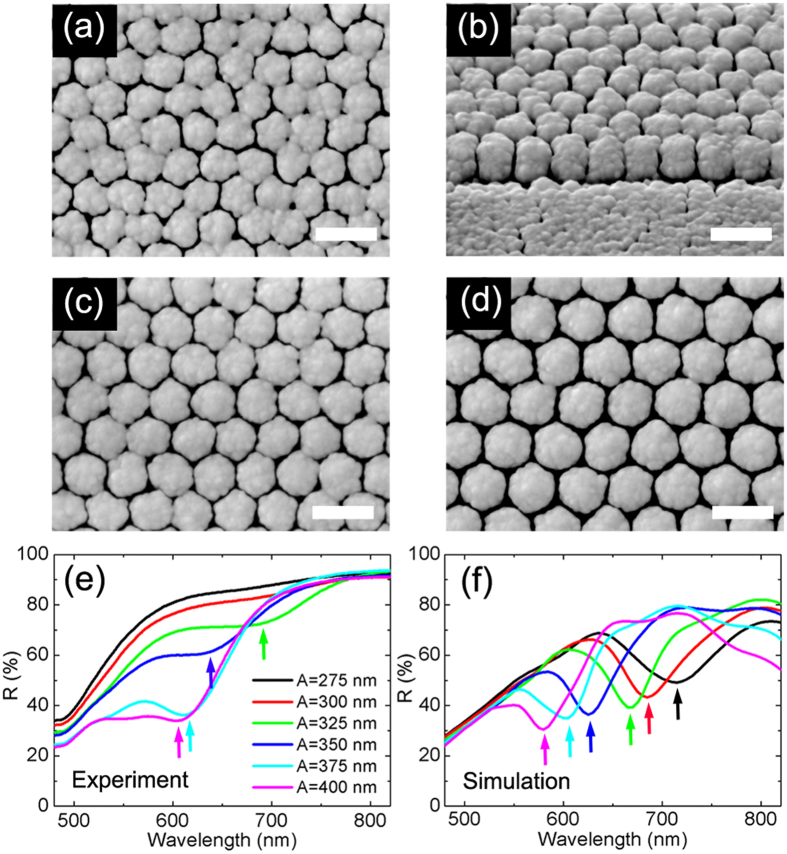
Fabricated nanoarrays and tunable reflectances. (**a–d**) SEM images of fabricated arrays with different array periodicities. Scale bars, 500 nm. (**a**) Top-view, *A* = 300 nm; (**b**) 45° oblique-view, *A* = 300 nm; (**c**) Top-view, *A* = 350 nm; (**d**) Top-view, *A* = 400 nm. (**e**) Experimental reflectance spectra. The positions of reflection dips for arrays *A* = 325 (green curve), 350 (blue curve), 375 (cyan curve) and 400 nm (magenta curve) are *λ* = 680, 632, 609 and 603 nm (indicated by the small arrows), respectively. (**f**) Corresponding FEM simulated reflectance spectra. The resonance wavelengths are 715, 685, 665, 625, 605 and 580 nm for arrays varying from *A* = 275 nm to *A* = 400 nm.

Despite the continuous progress of nanofabrication techniques, the production of nanostructure with reproducible and controllable nanogaps remains a challenge, especially for gap dimensions under 10 nm^8^,^20^,^38^,^39^,^40^,^41^,^42^. Experimentally, we have fabricated a series of periodic gold nanorod arrays with inter-rod gaps of sub-10 nm to 30 nm by combining electron beam lithography (EBL) with glancing angle deposition (GLAD) method^43^, as is shown in Fig. 2. This two-step method can fabricate closely spaced periodic nanorod arrays with good reproducibility and tunability. To be specific, two dimensional periodic hexagonal arrays of silicon columns (200 nm in diameter and 100 nm in height) were firstly prepared by EBL, and vertically aligned gold nanorods were then grown on the silicon template using GLAD^44^,^45^. See Methods for additional fabrication details. The height of the nanorods in our experiment is about 350 nm. The separation between silicon columns was tuned from 75 to 200 nm with 25 nm intervals. As a result, the array period *A* = *D* + *d* varies from 275 to 400 nm. The average inter-rod gap width is *d* = 8, 9, 10, 14, 20 and 28 nm, respectively. Meanwhile *D* increases with the pre-set *A* as well (See Supplementary Fig. S1a). For convenience, we use the period *A* to indicate different samples in the following characterizations.

### Reflectance measurements and simulations

Optical reflectances of these arrays were measured at normal incidence by angle-resolved microspectrometer (ARM62) with a linearly polarized light source. As is shown in Fig. 2e, pronounced and broad reflection dips are observed for the four arrays with largest period *A*. These dips are blue-shifted from about 680 to 600 nm as *A* (and *d*) increases. Modeled with geometric sizes (*d*, *D*, and *h*) of the arrays identical to those of the measured samples, 3D FEM simulations were performed. Figure 2f shows the simulated reflectance spectra, which are in good agreement with the measured ones. The simulated dips also exhibit a blue-shift from 715 to 580 nm as *A* increases from 275 to 400 nm. The spectral deviation between the experimental dips and the calculated ones is within 25 nm. These differences in the magnitude and resonance wavelength between calculated and experimental spectra can be mainly attributed to disparities between the modeled geometry and the actual one. In particular, for *A* = 275 and 300 nm, simulations of perfect lattice structures predict reflection dips while there is no observed dips in the experiment. This significant deviation is caused by the collapse of gap sidewalls and the failure of a free-standing lattice structure, which can be seen from the SEM images in Fig. 2a,b.

### SERS measurements

**Figure 3:**
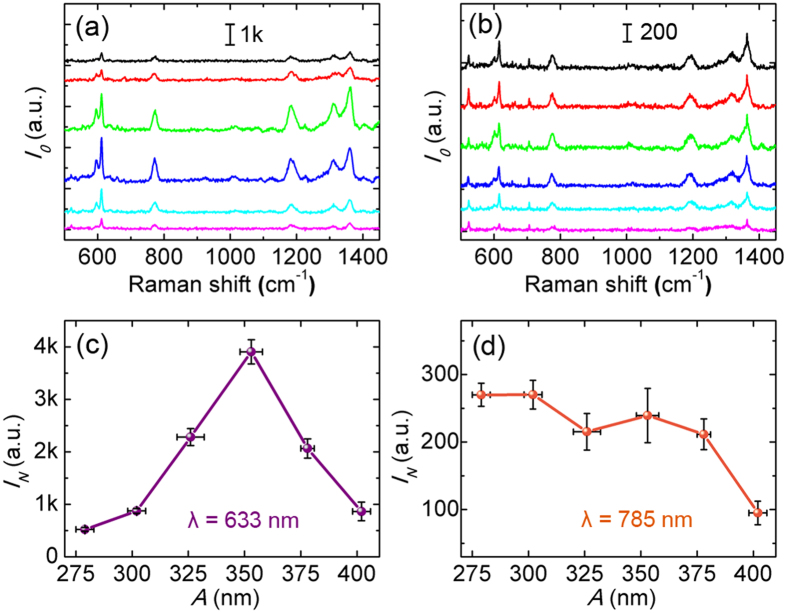
SERS characterization and normalization. (**a,b**) Typical SERS spectra with baseline subtracted of R6G (10^−6^ M) excited at 633 nm (left) and 785 nm (right). The color legend is the same as in Fig. 2e. (**c,d**) Normalized SERS intensity *I*_*N*_ of 612 cm^−1^ band. Error bars represent the standard deviation of *A* and the uncertainty of normalized SERS intensity.

For further understanding of the lattice coupling, near-field plasmonic properties were investigated. It is widely accepted that the electromagnetic enhancement factor (EF) of SERS is approximately proportional to |***E***/***E***_***0***_|^4^ based on its electromagnetic (EM) theory^31^,^46^. Experimentally, the SERS properties of the gold nanorod arrays were characterized at 633 nm and 785 nm laser wavelength, using 10^–6^ M aqueous solution of Rhodamine 6G (R6G) as the probe molecule. The molecular resonance is about 500–575 nm^47^. The laser polarization was set identically to that of the ARM62 light source. Typical measured SERS spectra with baseline subtracted are shown in Fig. 3a,b. Assuming that the probe molecules were arranged randomly and uniformly on the surface of the nanorods, the observed SERS intensity *I*_*0*_ was then proportional to the product of the total surface area *S* and the averaged SERS EF of single molecule. To get the information of the near-field enhancement, 612 cm^−1^ characteristic band was used for further analysis since its small Stokes shift limits errors associated with applying the |***E***/***E***_***0***_|^4^ approximation. Each SERS intensity *I*_*N*_ shown in Fig. 3c,d is the average of six measured intensities normalized by the total surface area of gold nanorods per unit area (See Methods and Supplementary Information). Usually the maximum near-field enhancement is associated with the minimum gap width for plasmonic structures^3^,^25^. However, in our experiment, the maximum *I*_*N*_ is obtained for *A* = 350 nm (*d* = 14 nm) at 633 nm laser excitation, which is nearly an order of magnitude higher than that for array *A* = 275 nm. On the other hand, when excited at 785 nm, the SERS intensity decreases almost monotonically as *A* (and *d*) increases (Fig. 3d).

## Discussion

### Simulations on near-field enhancement

Supposing the near-field and far-field plasmonic responses correlate with each other, the lattice coupling indicated by the reflection dip at 632 nm (Fig. 2e) for array *A* = 350 nm may thus be responsible for the above SERS behaviors. Yet the correlation can be argued as demonstrated in recent research works^28^,^31^. In practical applications, it has recently been fully appreciated that there exists a distinct deviation of spectral positions between the near- and far-field plasmonic responses as the near-field resonance is usually red-shifted compared to the far-field resonance, and in many cases only single frequencies are considered for near-field enhancement^25^,^48^,^49^,^50^,^51^,^52^. Here we apply a self-defined average near-field enhancement spectrum 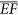 to address this point. The 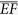 spectrum was obtained concurrently during FEM simulations by averaging the surface integral of field enhancement factor |***E/E***_0_|^4^ over the gold nanorod surface *S* that is exposed to air^31^,^46^:


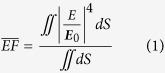


The physical significance of 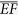 can be understood as the averaged electromagnetic EF of SERS as Raman probe molecules are arranged on the metal surface, leading to 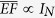.

**Figure 4:**
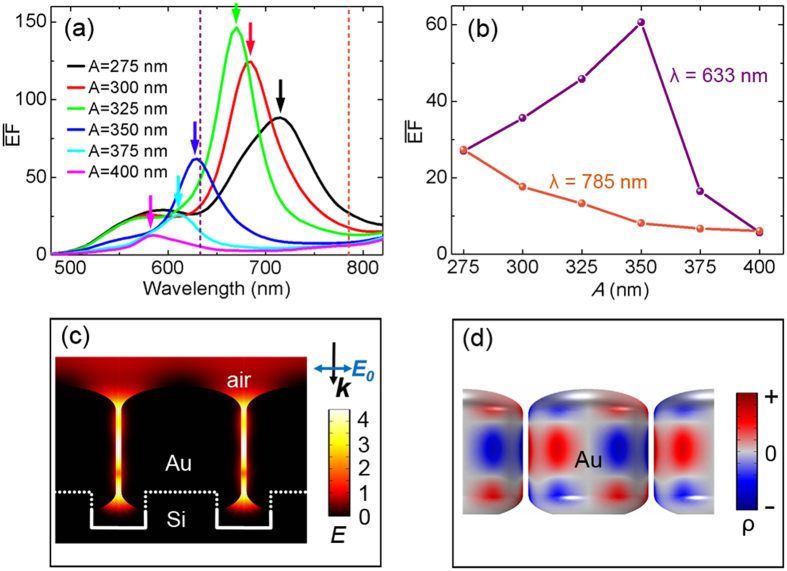
Near-field enhancement simulations. (**a**) FEM calculated near-field 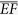 spectra for the same models as in Fig. 2f. (**b**) Extracted 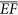 values at *λ* = 633 and 785 nm. (**c**) Logarithmic |***E***|^4^ distributions for array *A* = 350 nm at *λ* = 630. (**d**) Corresponding 3D surface charge distributions, indicating the lattice coupling of six-pole plasmon modes.

Figure 4a shows the calculated 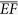 spectra. It is noticed that there is always a 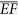 peak corresponding to the reflection dip at the same wavelength (See the small arrows in the spectra), illustrating the strong correlation between the near-field SERS enhancement and far-field reflectance for the lattice structures. When the maximum near-field enhancement is achieved, an enhanced interaction of the electromagnetic field with the lossy metal arises through the LSPRs, thus the absorption (converted to heat) is enhanced and the re-emission energy is reduced, leading to the reflection dip. Furthermore, the 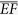 values at 633 nm and 785 nm are extracted and plotted in Fig. 4b. It turns out that the 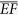 trends are consistent very well with experimental SERS intensity at 633 nm and 785 nm (Fig. 3c,d). Near-field electric field distributions (in the form of logarithmic |***E/E***_0_|^4^) of array *A* = 350 nm when the 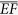 value reaches the maximum at λ = 630 nm is plotted in Fig. 4c. As expected, local electric fields in the gaps are strongly enhanced. Through the mapping of 3D surface charge distributions at the resonance wavelengths (Fig. 4d), we confirmed the resonances of different arrays in the wavelength range of 600–700 nm to be the lattice coupling of six-pole plasmon modes. Seen from Fig. 4a, it can further be predicted that when excited by 660 nm laser, array *A* = 325 nm will exhibit a much stronger SERS intensity instead.

### Effects of surface roughness

Noticing that the maximum |***E***/***E***_***0***_|^4^ in Fig. 4c is about 3 × 10^4^, it may not be strong enough to maintain observable SERS signals in the experiment. Actually, gold nanorods are roughened by raised particles present on the top and side surfaces, owing to shadowing effects and intrinsic atomic diffusion during GLAD^43^. Since its discovery in the 1970s, the surface roughness has always been playing an important role in SERS^32^,^33^,^53^. To consider the roughness effect, a computational “rough” model was built by adding six small particles distributed symmetrically on the middle side of the smooth nanorods (See Methods).

**Figure 5:**
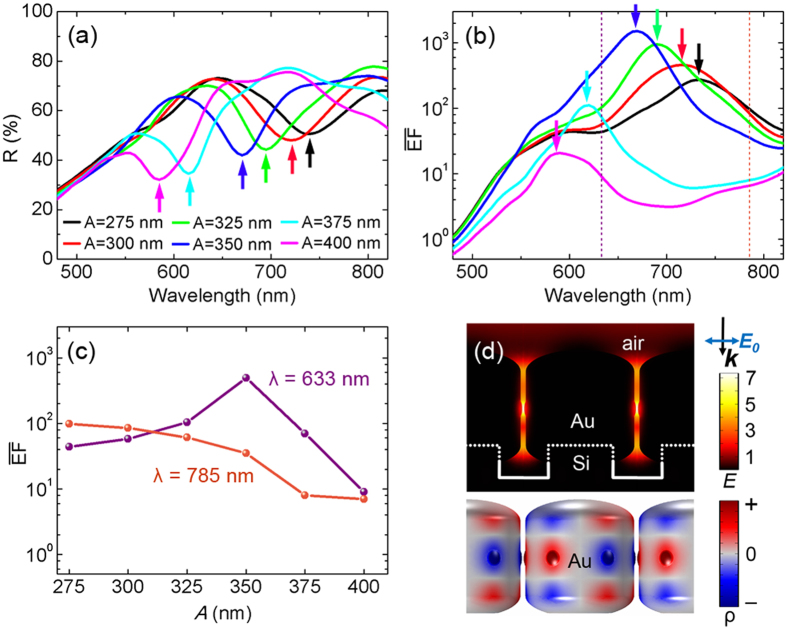
Simulation results of rough models. (**a**) Far-field reflectance spectra. (**b**) Near-field 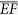 spectra. (**c**) Extracted 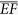 values at *λ* = 633 and 785 nm. (**d**) Logarithmic |***E***|^4^ distributions (upper) and corresponding surface charge distributions (bottom) of array *A* = 350 nm at resonance wavelength *λ* = 670 nm.

As is shown in Fig. 5a, the calculated reflection dips of rough models are 5–35 nm red-shifted relative to that of the corresponding smooth ones (Fig. 2f). Figure 5b shows the calculated 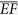 spectra for rough models, whose 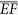 peaks are also slightly red-shifted with respect to that of smooth models. It is worth mentioning that the 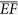 intensities of the former are nearly an order of magnitude higher now. The extracted 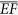 values at *λ* = 633 and 785 nm (Fig. 5c) show the same trend as the experimental SERS enhancement in Fig. 3c,d. In this case, maximum local |***E***/***E***_***0***_|^4^ reaches up to 2 × 10^7^ at *λ* = 670 nm while the lattice coupling of six-pole plasmon modes is sustained (Fig. 5d). It is now clear that at 633 nm, the lattice coupling of six-pole plasmon modes in array *A* = 350 nm is strongly excited and hence the maximum SERS enhancement is obtained. For the case with *λ* = 785 nm excitation, the gradual decoupling of this mode yields weakened SERS signals as *A* increases.

## Conclusion

In conclusion, we have demonstrated, both experimentally and theoretically, the lattice coupling of multipole plasmon modes for the attractive lattice structure: closely spaced gold nanorod arrays. We confirm the plamon modes directly through the mapping of 3D surface charge distributions, offering a new insight into the higher order cavity modes coupled with each other in the lattice. The lattice coupling can be greatly tuned by changes in inter-rod gaps and nanorod heights, while the influence of the nanorod diameter is almost negligible to some extent. Experimentally, we have fabricated this kind of lattice structures using EBL followed by GLAD. For the far-field response, a series of tunable and pronounced reflection dips are observed, indicating an efficient coupling of the lattice to the incident light. Meanwhile, the near-field response of the lattice, in terms of SERS, are found to be further enhanced by nearly an order of magnitude when excited at the resonance wavelength. It is then demonstrated that the near-field and far-field responses correlate well with each other, which is of significant importance for maximizing the near-field enhancement at a specific excitation wavelength. 3D FEM simulations on both the near-field and far-field properties are in good coincidence with the experimental results. As an active plasmonic platform, closely spaced metallic nanoarrays is of broad interest not only for SERS but also for other plasmonic applications, such as subwavelength imaging or metamaterials.

## Methods

### FEM modeling

3D electrodynamics simulations were performed using the finite element method (FEM)^46^,^54^ in COMSOL Multiphysics software package^55^ (installed on a Quad Intel Xeon CPU, 64 GB RAM workstation). The optical constants of silicon substrates were evaluated by a quadratic interpolation of published values in Palik’s book^56^ while a Lorentz-Drude dispersion model was used to fitting the dielectric function of gold^57^:


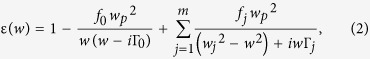


where *w*_*p*_ is the plasma frequency with oscillator strength *f*_*0*_ and damping constant *Γ*_*0*_. The last term of Eq. 1 is the result of the Lorentz modification, where *m* is the number of oscillators with frequency *w*_*j*_, strength *f*_*j*_ and damping constant *Γ*_*j*_. The fitting parameter values are *f*_*0*_ = 0.760, *w*_*p*_ = 9.03 eV, *Γ*_*0*_ = 0.053 eV, *f*_*1*_ = 0.024, *Γ*_*1*_ = 0.241 eV, *w*_*1*_ = 0.415 eV, *f*_*2*_ = 0.010, *Γ*_*2*_ = 0.345 eV, *w*_*2*_ = 0.830 eV, *f*_*3*_ = 0.071, *Γ*_*3*_ = 0.870 eV, *w*_*3*_ = 2.969 eV, *f*_*4*_ = 0.601, *Γ*_*4*_ = 2.294 eV, *w*_*4*_ = 4.304 eV, *f*_*5*_ = 4.384, *Γ*_*5*_ = 2.214 eV, *w*_*5*_ = 13.32 eV.

Each end of the gold nanorod is capped with a semi-ellipsoid (minor axis/major axis = 0.4). The silicon template consists of two dimensional periodic hexagonal arrays of silicon columns (200 nm in diameter and 100 nm in height), which can be fabricated by electron beam lithography (EBL). The bottom Si substrate is set to be semi-infinite during the simulation. Periodic boundary conditions are loaded onto the hexagonal unit to simulate infinite periodic gold hexagonal nanorod arrays. Using adaptive meshing, the highest spatial resolution of the grid is ~0.5 nm in the simulation.

The reflectance *R* is calculated by integrating the Poynting vector 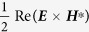 on an auxiliary surface *S* in the reflecting areas:


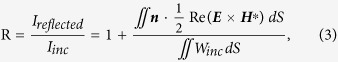


where *I*_*reflected*_ and *I*_*inc*_ are the reflected and incident intensity respectively, ***n*** is the normal vector pointing outwards from the arrays (thus the second term on the right side of the equation is a negative value), ***E*** and ***H*** are the total electric and magnetic field respectively, 
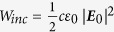
 is the power flow per unit area of the incident plane wave, |***E***_***0***_| = 1 V/m is the incident electric field, *c* is the velocity of light and ε_0_ is the permittivity of vacuum. The computational time for an entire spectrum (i.e. ~90 spectral points in the wavelength range of 480–820 nm) is ~36 h.

To consider the roughness effect, a computational “rough” model was built by adding six small particles distributed symmetrically on the middle side of the smooth nanorod. The height of the particles is 5 nm when *A* ≥ 350 nm resulting in 18, 10, 4 nm gaps between adjacent nanorods for arrays *A* = 400, 375 and 350 nm respectively. And the particle is squashed when *A* ≤ 350 nm to maintain a hot spot of 4 nm gap so as to avoid artificially excessive strong near-field couplings, as SERS enhancement is rather sensitive to the separation of narrow gaps. As a result, the gaps are narrowed correspondingly to 4, 4, 4, 4, 10 and 18 nm from 8, 9, 10, 14, 20 and 28 nm in the local regions compared with the smooth models. Raised particles in different models remain 40 nm in diameter.

### 3D plasmon mapping

The induced surface charge density is considered over the whole metal structures. Based on the skin effect, we assume that the induced charge density *ρ*_*r*_ is the largest at the metal surface *S* and decreases exponentially when spreading into the metal:


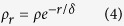


where *ρ* is the charge density at the surface, *r* is the depth from the surface and *δ* is the skin depth^58^,^59^,^60^. The total polarization charge *Q* within the gold nanorod is thus:


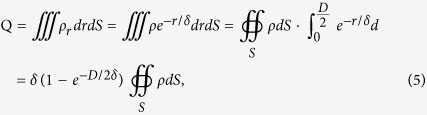


where *D* is the diameter of the nanorod. On the other hand, the Gauss’ law in the integral form:


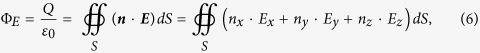


where Φ_*E*_ is the electric flux through the metal surface *S*, *ε*_*0*_ is the permittivity of vacuum,***n*** = (*n*_*x*_*, n*_*y*_*, n*_*z*_) is the outward normal vector of the metal surface and ***E*** = (*E*_*x*_, *E*_*y*_, *E*_*z*_) is the local electric field. The surface charge density can then be deduced by:


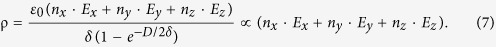


In the process of FEM calculations and plasmon mapping, (*n*_*x*_*∙E*_*x*_ + *n*_*y*_*∙E*_*y*_ + *n*_*z*_*∙E*_*z*_) is used to indicate the surface charge density *ρ*. The use of this mapping approach makes it possible for us to acquire directly 3D surface charge distributions, which is ideally suited to recognize the geometry (or order) of complicated and hybridized plasmon modes^25^,^26^.

### Sample fabrication

The area of each lithographically defined silicon column array was 200 μm × 200 μm. During GLAD, the gold vapor flux angle was set to 88°. The substrate holder was cooled to ~20 °C by a house-designed liquid nitrogen cooling system. The background vacuum of the chamber was below 2 × 10^−5^ Pa. The substrate holder rotated at a speed of 2 rpm, and the deposition rate, about 0.5 nm/s, was monitored by a quartz crystal microbalance (QCM). All samples were characterized by scanning electron microscopy (FEI Quanta 200 FEG) operated at 15 kV. Supplementary Fig. S1a shows the averaged diameter *D* of the nanorods in each array. As *A* increases from 275 to 400 nm, the inter-rod gap *d* = *A* − *D* = 8, 9, 10, 14, 20 and 28 nm, respectively.

### SERS characterization

Each sample was dipped in 1 × 10^−6^ M aqueous solution of R6G for 30 minutes and dried with a nitrogen stream. The averaged Raman spectrum of R6G was obtained by measuring and averaging the spectra from six different areas in each array. The averaged SERS intensities *I*_*0*_’ of 612 cm^−1^ band are plotted in Supplementary Fig. S2. The measurements were performed with a Reinshaw 100 Raman spectrometer using a 633 nm He-Ne laser as the excitation source, with the spot size of the laser beam defocused to about 10 μm in diameter, and the laser power of 0.47 mW, signal accumulation time of 10 second per 600 cm^−1^, 10× objective and NA = 0.25. For the Raman spectra excited by 785 nm laser, a Horiba YJ HR-800 Raman spectrometer using a 785 nm semiconductor laser as the excitation source was used, with the spot size of the laser beam defocused to about 3 μm in diameter, the laser power of 10 mW, signal exposure time of 10s, 50× objective and NA = 0.5.

To estimate the surface area, both the primary nanorod structure (smooth model) and the raised little particles at the surface (surface roughness) are taken into account (Supplementary Fig. S1d). The number of raised particles are counted from SEM images. To consider the effect of all the raised particle on the total surface area, distributions of the diameter of particles in array *A* = 400 nm is shown in Supplementary Fig. S1b,c as an example of our measurement process. The distribution follows a normal distribution. The total surface area of gold nanorods per unit area is the specific surface area estimated over 1 × 1 μm^2^ vertically projected region on the sample. If theses surface areas are normalized by that of array *A* = 275 nm (It is 9.36 μm^2^), then we get 1.00 ± 0.04, 0.86 ± 0.03, 0.78 ± 0.04, 0.69 ± 0.03, 0.65 ± 0.02 and 0.62 ± 0.02 respectively as *A* increases from 275 to 400 nm, which is defined as the surface area factor for the normalizations. The normalized SERS intensity *I*_*N*_ is the averaged SERS intensity *I*_*0*_’ (Supplementary Fig. S2) divided by the surface area factor.

## Additional Information

**How to cite this article**: Huang, Y. *et al.* Tunable Lattice Coupling of Multipole Plasmon Modes and Near-Field Enhancement in Closely Spaced Gold Nanorod Arrays. *Sci. Rep.* **6**, 23159; doi: 10.1038/srep23159 (2016).

## Supplementary Material

Supplementary Information

Supplementary Movie S1
