# Supplementary material for: Tunable Lattice Coupling of Multipole Plasmon Modes and Near-Field Enhancement in Closely Spaced Gold Nanorod Arrays
Source: Sci Rep. 2016 Mar 17;6:23159. doi: 10.1038/srep23159 (PMC4794719; doi:10.1038/srep23159)
Supplement: Supplementary Information [file srep23159-s1.pdf]

Supplementary Information for:

Tunable Lattice Coupling of Multipole Plasmon Modes and Near-Field Enhancement in Closely Spaced Gold Nanorod Arrays

Yu Huang[1,*], Xian Zhang[1,*], Emilie Ringe[2], Mengjing Hou[1], Lingwei Ma[1] & Zhengjun Zhang[3]

[1]State Key Laboratory of New Ceramics and Fine Processing, School of Materials Science and Engineering, Tsinghua University, Beijing 100084, P. R. China.

[2]Deparment of Materials Science and Nanoengineering & Laboratory for Nanophotonics, Rice University, 6100 Main Street, Houston, TX 77005, USA.

[3]Key Laboratory of Advanced Materials (MOE), School of Materials Science and Engineering, Tsinghua University, Beijing 100084, P. R. China.

[*]These authors contributed equally to this work. Correspondence and requests for materials should be addressed to Z.J.Z. (email: zjzhang@tsinghua.edu.cn).

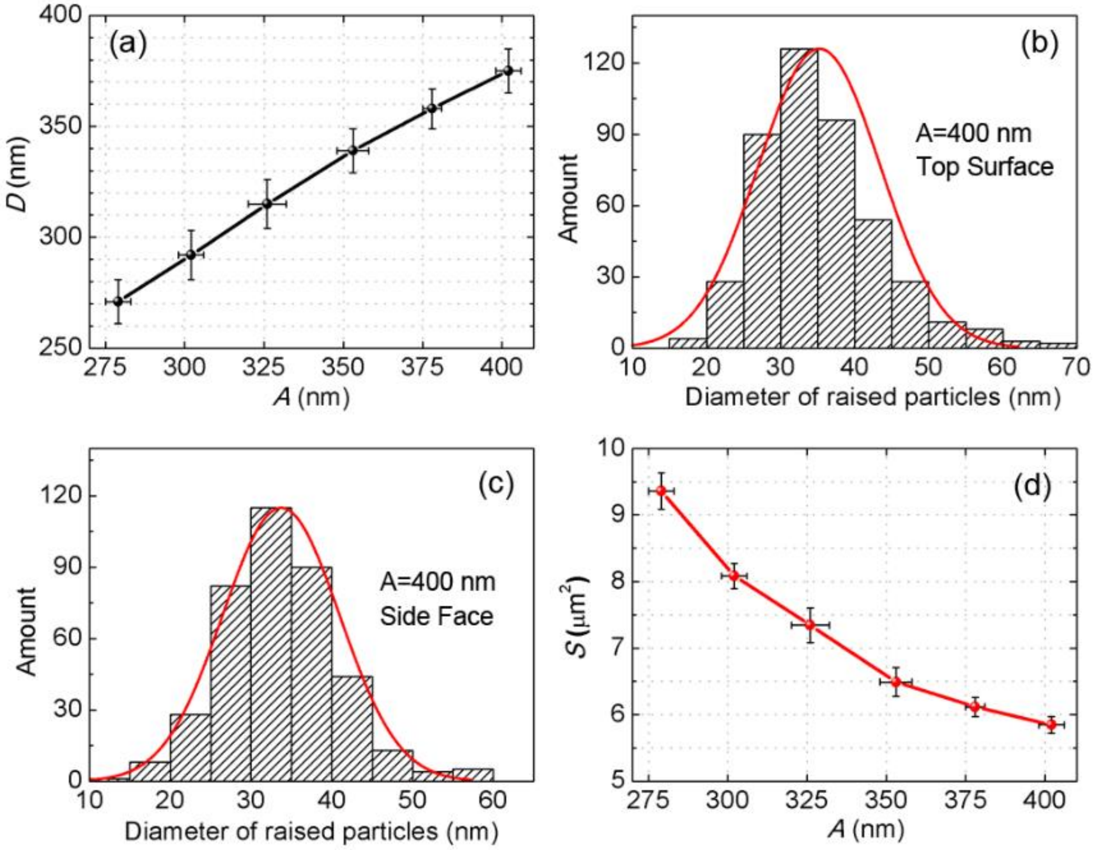

Supplementary Figure S1 | Sizes of fabricated gold nanorod arrays. (**a**) Diameter *D* of the nanorod in fabricated arrays. *A* is the pre-set array periodicity. Error bars are the standard deviations. (**b,c**) Distributions of the diameter of raised particles on the top surface and side face in array *A* = 400 nm. (**d**) Estimations of the total surface area of the nanorods on 1× 1 μm^2. Error bars are respectively uncertainties of total surface area *S* and standard deviations of *A*.

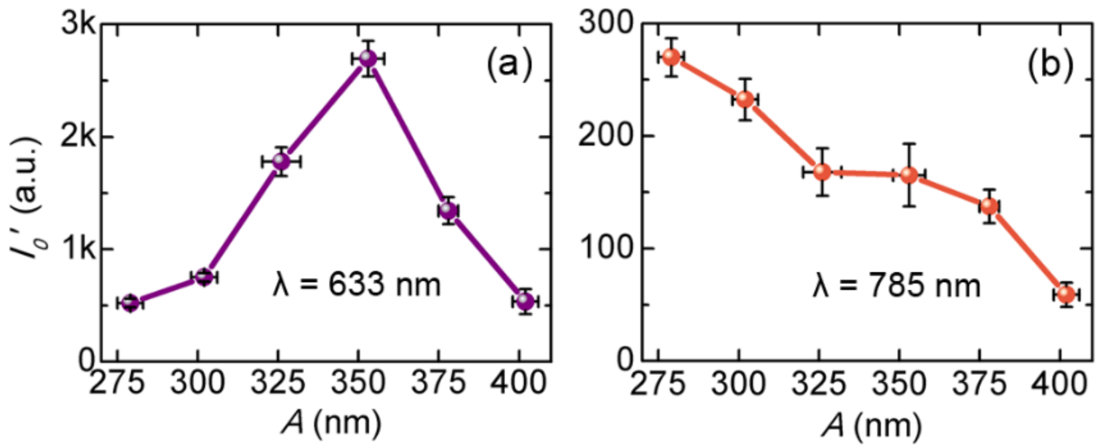

Supplementary Figure S2 | Average SERS intensity I_0' of 612 cm^{-1} band. (**a**) 633 nm excitation. (**b**) 785 nm excitation. Error bars are the standard deviations.

Supplementary Movie S1 | Three dimensional surface charge distributions within one oscillation for Fig. 1e: array h = 300 nm, D = 300 nm, d = 10 nm, λ = 675 nm, the lattice coupling of six-pole plasmon modes.
